# Satisfaction with life among university students from nine countries: Cross-national study during the first wave of COVID-19 pandemic

**DOI:** 10.1186/s12889-021-12288-1

**Published:** 2021-12-11

**Authors:** Aleksandra M. Rogowska, Dominika Ochnik, Cezary Kuśnierz, Monika Jakubiak, Astrid Schütz, Marco J. Held, Ana Arzenšek, Joy Benatov, Rony Berger, Elena V. Korchagina, Iuliia Pavlova, Ivana Blažková, Zdeňka Konečná, Imran Aslan, Orhan Çınar, Yonni Angel Cuero-Acosta

**Affiliations:** 1 Institute of Psychology, University of Opole, 45-052 Opole, Poland; 2 Faculty of Medicine, University of Technology, 40-555 Katowice, Poland; 3 Faculty of Physical Education and Physiotherapy, Opole University of Technology, 45-758 Opole, Poland; 4 Faculty of Economics, Maria Curie-Sklodowska University in Lublin, 20-031 Lublin, Poland; 5 Department of Psychology, University of Bamberg, 96047 Bamberg, Germany; 6 Faculty of Management, University of Primorska, 6101 Koper, Slovenia; 7 Department of Special Education, University of Haifa, 3498838 Haifa, Israel; 8 The Center for Compassionate Mindful Education, 69106 Tel Aviv, Israel; 9 Bob Shapell School of Social Work, Tel-Aviv University, 69978 Tel Aviv, Israel; 10 St. Petersburg School of Economics and Management, HSE University, 194100 St. Petersburg, Russia; 11 Institute of Industrial Management, Economics and Trade, Peter the Great St. Petersburg Polytechnic University, St. Petersburg, 195251 Russia; 12 Department of Theory and Methods of Physical Culture, Lviv State University of Physical Culture, Lviv, 79007 Ukraine; 13 Department of Regional and Business Economics, Mendel University in Brno, 613 00 Brno, Czech Republic; 14 Faculty of Business and Management, Brno University of Technology, 612 00 Brno, Czech Republic; 15 Health Management Department, Bingöl University, 12000 Bingöl, Turkey; 16 Faculty of Economics and Administrative Sciences, Ataturk University, 25240 Erzurum, Turkey; 17 School of Management, Universidad del Rosario, Bogotá, PC 111711 Colombia

**Keywords:** Cross-national study, University and college students, Satisfaction with life, Exposure to COVID-19, Perceived negative impact of coronavirus on life, Physical activity, Gender differences

## Abstract

**Background:**

A cross-sectional study was performed to examine life satisfaction differences between university students from nine countries during the first wave of the COVID-19 pandemic. A cross-national comparison of the association between life satisfaction and a set of variables was also conducted.

**Methods:**

Participants in the study were 2349 university students with a mean age of 23 years (*M* = 23.15, *SD* = 4.66). There was a predominance of women (69.26%) and individuals studying at the bachelor level (78%). The research was conducted between May and July 2020 in nine countries: Slovenia (n=209), the Czech Republic (Czechia)(n=308), Germany (n=267), Poland (n=301), Ukraine (n=310), Russia (n=285), Turkey (n=310), Israel (n=199), and Colombia (n=153). Participants completed an online survey involving measures of satisfaction with life (SWLS), exposure to COVID-19, perceived negative impact of coronavirus (PNIC) on students' well-being, general self-reported health (GSRH), physical activity (PA), and some demographics (gender, place of residence, level of study). A one-way ANOVA was used to explore cross-national differences in life satisfaction. The χ^2^ independence test was performed separately in each country to examine associations between life satisfaction and other variables. Bivariate and multivariate logistic regressions were used to identify life satisfaction predictors among a set of demographic and health-related variables in each of the nine countries.

**Results:**

The level of life satisfaction varied between university students from the nine countries. The results for life satisfaction and the other variables differed between countries. Numerous associations were noted between satisfaction with life and several variables, and these showed cross-national differences. Distinct predictors of life satisfaction were observed for each country. However, poor self-rated physical health was a predictor of low life satisfaction independent of the country.

**Conclusions:**

The association between life satisfaction and subjective assessment of physical health seems to be universal, while the other variables are related to cross-cultural differences. Special public health attention should be focused on psychologically supporting people who do not feel healthy.

**Supplementary Information:**

The online version contains supplementary material available at 10.1186/s12889-021-12288-1.

## Background

Satisfaction with life is the global method of cognitively self-judging well-being across a broad set of factors relating to human life, including work, family, physical and mental health, and the sexual sphere [1]. Research has shown that life satisfaction is one of the most important indicators of successful adaptation to life, and it can be beneficial for health, longevity, and social relationships [2, 3]. Among various factors that may be associated with high life satisfaction, income, job satisfaction, needs satisfaction, resilience, and social relationships and support have been identified as positive predictors, whereas unemployment, stress, anxiety, and depression have been shown to decrease life satisfaction [4–17].

An infectious disease known as severe acute respiratory syndrome coronavirus-2 (SARS-Cov-2) was first reported in Wuhan (China) in early December 2019 and then spread rapidly around the world [18, 19]. The first wave of the coronavirus pandemic (COVID-19) appeared globally in February 2020 and lasted until the middle of the year. Many countries introduced a period of lockdown that involved various levels of restrictions. Depending on the country, these involved travel, shopping, gatherings, separation from friends and family, physical exercise, and access to critical public services including medical and social services, childcare, and education. A national quarantine caused a rapid transformation of the education system worldwide, from traditional stationary learning to remote online learning, and this was a significant stress source for university students and teachers [20–23]. Difficulties with online learning included unstable internet connections, the need for additional investment in a greater internet limit, increased struggles with time management, and problems with focusing while learning online [24]. Stress was also related to the loss of social gatherings.

A review study [25] demonstrated that coronavirus-related lockdowns were associated with many adverse psychological effects, such as increases in negative emotions, insomnia, stress, anxiety, and depression, leading to declines in work performance and family functioning and increases in financial problems. The study also showed the adverse impact of the COVID-19 pandemic on sexual life, particularly among women [26–28]. Research performed in many countries indicated that the COVID-19 pandemic decreased mental health and life satisfaction [29–39]. However, comparative studies between countries are lacking. We studied nine countries during the first wave of the COVID-19 pandemic, compared the results, and identified predictors of life satisfaction in university students.

Among the various predictors of well-being during the COVID-19 pandemic, female gender was found to be related to lower satisfaction with life and higher levels of stress, anxiety, and depression [39–48]. Current good health and high exercise status affected life satisfaction during the lockdown [38, 39, 42, 43, 49–51]. Eek et al. [52] showed that people who reported decreased physical activity (PA) levels during the COVID-19 pandemic reported low levels of life satisfaction and aerobic capacity. Moreover, adults who exercise more have a lower risk of being infected with coronavirus [53]. In the Spanish population, Gonzalez-Bernal et al. [54] recently associated high life satisfaction with fewer days of home confinement, the perception of having received enough information about COVID-19 and its prevention, having private access to the outside, being employed, being male, and not experiencing isolation. Also, a high level of work engagement and a low level of family conflict may be predictors of high satisfaction with life [55].

Studies performed during the COVID-19 pandemic showed significant decreases in well-being and life satisfaction in particular [39, 42–45, 56–63]. However, predictors of satisfaction with life during the coronavirus pandemic, in particular among the university student population, have not been identified. There is also a lack of international research comparing the level of life satisfaction among countries at the same point in the pandemic, namely during the first wave of its outbreak. This study compares university students' satisfaction with life in the following nine countries: Slovenia, the Czech Republic (Czechia), Germany, Poland, Ukraine, Russia, Turkey, Israel, and Colombia. Those countries differ significantly in terms of well-being, as indicated by the World Happiness Report 2021 based on the Gallup World Poll survey [64]. According to that report, these countries were ranked in the following positions among 149 countries in 2020 during the COVID-19 pandemic: Germany (rank 7), Israel (rank 11), Czechia (rank 16), Slovenia (rank 26), Poland (rank 39), Colombia (rank 55), Russia (rank 60), Ukraine (rank 69), and Turkey (rank 78). However, we expect more nuanced differences among the university student population. Even though students are a socially privileged population, they have a higher risk of developing depression than the general population, and this was the case even in the pre-pandemic period [65, 66].

Concurrently, we examined predictors of life satisfaction such as gender, place of residence, level of study, physical activity, exposure to the COVID-19 pandemic, perceived negative impact of COVID-19 on students' well-being (including qualifications, economic status, and social relationships), and physical health.

## Methods

### Study design and procedure

A cross-national online survey study was performed during the first wave of the COVID-19 pandemic, between May and July 2020, in nine countries: Slovenia (May 14–26), the Czech Republic (June 17–July 24), Germany (2–25 June), Poland (May 19–June 25), Ukraine (May 14–June 2), Russia (1–22 June), Turkey (16–29 May), Israel (May 21–June 3), and Colombia (June 5). Students were recruited from the following universities: Maria Curie-Sklodowska University in Lublin (Poland, n = 148), University of Opole (Poland, n = 152), University of Primorska in Koper (Slovenia, n = 209), Mendel University in Brno (Czech Republic, n = 310), Lviv State University of Physical Culture (Ukraine, n = 310), Peter the Great St. Petersburg Polytechnic University (Russia, n = 155), Higher School of Economics (Russia, n = 90), St. Petersburg State University of Economics and Finance (Russia, n = 42), University of Bamberg (Germany, n = 270), University of Haifa (Israel, n = 199), Bingöl University (Turkey, n = 148), Atatürk University (Turkey, n = 110), Muğla Sıtkı Koçman University (Turkey, n = 35); Ağrı İbrahim Çeçen University (Turkey, n = 6); Fırat University (Turkey, n = 3); Kırıkkale University (Turkey, n = 1), Adnan Menderes University (Turkey, n = 1), Başkent University (Turkey, n = 3), Boğaziçi University (Turkey, n = 1), Dicle University (Turkey, n = 1), and Istanbul University (Turkey, n = 1), Del Rosario University (Colombia, n = 142), and El Bosque University (Colombia, n = 13, 8%). The survey was created and distributed via Google Forms in all countries, except for Germany where SoSci Survey was used. To meet cross-cultural adaptation standards [67, 68], partners from various countries translated survey questions from English into the original languages. The invitations to the online questionnaire were disseminated to students via e-mail, social media (e.g., Facebook, Viber, LINK, Telegram), and e-learning platforms (e.g., Moodle, MS Teams). In some countries, researchers also collaborated with student trade unions and student government organizations to disseminate invitations to participate in the study among students from target universities. The eligibility criterion was being a college or university student. All respondents were eligible for inclusion in the research and confirmed their student status by answering the question about their current year and field of study. The participants completed the questionnaire within 23 minutes on average (*M* = 23.26; *SD* = 44.03). To minimalize sources of bias, we collected a diverse student sample in terms of its key characteristics: type of university, field of study, and study cycle.

The University Research Ethics Committee (KEBN) at the University of Opole approved the study protocol (no. 1/2020). Students were assured about the anonymity and confidentiality of the survey and were informed that they could pull out of the study whenever they wanted. Informed consent was obtained from all participants, since information about the study and informed consent were included in the first section of the questionnaire. The study was conducted according to the 1964 Helsinki Declaration and its later amendments or comparable ethical standards (like the APA ethical standards). In seven countries, no compensation was offered to students or researchers as an incentive to participate. In Germany, however, university students were able to register for the chance to win a 20 EUR Amazon gift card, if they participated. Israeli students could win a 300 NIS gift card. No specific funding was given to scientists for the research and manuscript preparation. This study is part of an international research project "Well-being of undergraduates during the COVID-19 pandemic: International study" and is registered at the Center for Open Science (OSF) [69].

### Measures

The **Satisfaction with Life Scale (SWLS)** measures global cognitive judgments regarding satisfaction with one's life [1, 2]. The scale consists of five items, to which respondents indicate how much they agree or disagree using a seven-point Likert scale (from 7 = *Strongly agree*, to 1 = *Strongly disagree*). Total scores ranged from 5 to 35, where the lowest scores indicate that a person is *Extremely dissatisfied* (scores between 5 and 9), scores ranging from 10 to 14 indicate *Dissatisfied*, 19-19 indicate *Slightly dissatisfied*, 20 indicates *Neutra*l, 21-25 indicate *Slightly satisfied*, 26-30 indicate *Satisfied*, and 31-35 indicate *Extremely satisfied*. For the purpose of the further analysis using the χ^2^ independence test and logistic regression, we dichotomized the SWLS scores into Lower (SWLS < 20, coded as 0) and Higher (SWLS > 21, coded as 1). The SWLS has shown satisfactory psychometric properties, a high level of internal consistency (Cronbach's α ranging from 79 to 89 in various studies), and high temporal reliability [3]. In the current study, Cronbach's α was 0.85 for the total sample and ranged between 0.60 and 0.86 in samples from particular countries (see Table 2 for more details).

**Exposure to COVID-19** [43] was assessed by eight questions about the consequences of the coronavirus, including infection, coronavirus testing, hospitalization for coronavirus, being in strict quarantine for at least 14 days, coronavirus infection in a close family member, die of the relative, job loss, and deteriorating economic status due to the effects of the COVID-19 pandemic. Individuals answered each of these questions (1 = *Yes*, 0 = *No*). A higher coronavirus exposure was related to a higher total score as a sum of eight items. Outcomes were classified into two categories: Lower exposure (score of 0, coded as 0) and Higher exposure (scores of 1-8, coded as 1).

The **Perceived Negative Impact of the Coronavirus (PNIC)** [43] on the students' well-being was measured using five statements. Participants answered each item using a 5-item Likert scale (from 1 = *I strongly disagree*, to 5 = *I definitely agree*) to express their level of fear regarding how the current situation related to the COVID-19 pandemic may negatively affect their life in each of the following five areas: (1) Completing the semester and qualifications; (2.) Finding a job and professional development; (3.) Financial situation; (4.) Relationships with loved ones and family; and (5.) Relationships with colleagues and friends. A total perceived negative impact of the coronavirus (PNIC) score was a sum of all items. Higher scores have been interpreted as the more significant impact of the coronavirus on students' lives. We used the median to dichotomize the total scores for the PNIC and its three subscales: Qualifications, Economic Status, and Social Relationships. For further statistical tests, the total PNIC was coded as follows (for the χ^2^ independence test and logistic regression): Lower (PNIC < 15, coded as 0); Higher (PNIC > 16, coded as 1). We added the scores of items 1 and 2 to form the Qualifications scale (then coded them as 0 = Lower, scores 2-6; 1 = Higher, scores 7-10), items 4 and 5 to form the Social Relationships scale (coded as 0 = Lower, scores 2-4; 1 = scores 5-10), and item 3 alone was used for the Economic Status scale (coded as 0 = scores 1-3; 1 = scores 4-5). The internal reliability of the scale across all countries was assessed with Cronbach's α and a score of 0.71 was obtained.

The **General Self-Rated Health (GSRH)** is a shorter alternative to the standard general health survey (SF-12V) [70, 71]. The GSRH contains two items. The first question concerned overall physical health (GSRH-1) (i.e., "In general, would you say your health is…?"), while the second, was a self-comparison of health with other people (i.e., "Compared to others your age, would you say your health is…?") (GSRH-2, Comparative). Both GSRH items were rated on a 5-point Likert scale (1 = *Excellent*, 2 = *Very Good*, 3 = *Good*, 4 = *Fair*, and 5 = *Poor*). Therefore, higher scores indicate a worse health status. Higher mortality was found among those who had poorly assessed their health using GSRH-1 [70]. We spilt the GSRH scores as follows (for the χ^2^ independence test and logistic regression): Better health (GSRH < 3, code 0); Worst health (GSRH > 4, coded as 1). The reliability coefficient for GSRH was Cronbach's α = 0.88 (*N* = 2,349) in the present study.

**Physical activity (PA**) [43] was evaluated using the following question "How many days a week did you exercise physically or pursue sports activities at home or away from home, at the university, in clubs, or at the gym, in the last month?". Participants marked their answer on an eight-point scale (from 0 = *Not one day* to 7 = *Seven days a week*). Next, the students evaluated how many minutes per day (on average) they spent on practice (minutes per day of PA). To calculate the previous week's PA level, the number of days was multiplied by the number of minutes per day. The participants were divided into two groups based on the PA total score, Sufficient (PA > 150 minutes weekly) and Insufficient (PA < 150 minutes weekly), according to the WHO recommendation [72].

**Demographic data** [69] included questions about age (number of years), gender (female, male), place of residence (village, town, city, agglomeration/metropolis), and current level of study (Bachelor, Master). We divided the answers regarding the place of residence into two categories (for the χ^2^ independence test and logistic regression): the first included students living in a village or town and the second included those living in a city, agglomeration, or metropolis.

### Participants

Initially, data collection included 2453 people, but 104 students (4.24% of the total sample) declined to participate. The final total sample included 2349 university students from the following nine countries: Slovenia (*n* = 209, 8.90%), the Czech Republic (*n* = 308, 13.20%), Germany (*n* = 267, 11.49%), Poland (*n* = 301, 12.81%), Ukraine (*n* = 310, 13.20%), Russia (*n* = 285, 12.13%), Turkey (*n* = 310, 13.20%), Israel (*n* = 199, 8.47%), and Colombia (*n* = 153, 6.60%). The mean age of university students was 23 years, with a range from 18 to 60 (*M* = 23.15, *SD* = 4.66). Most of the students were female (*n* = 1627, 69.26%), lived in a village/town (1284, *n* = 54.66), and were studying at the bachelor level (*n* = 1843, 78.45%). The distribution of demographics with regard to each particular country is shown in Table 1.

**Table 1:** Demographics of the university students from the nine countries

		Total (*N* = 2349)		Slovenia (*n* = 209)		Czechia (*n* = 310)		Germany (*n* = 270)		Poland (*n* = 301)		Ukraine (*n* = 310)		Russia (*n* = 285)		Turkey (*n* = 310)		Israel (*n* = 199)		Colombia (*n* = 153)	
Variable		*n*	%	*n*	%	*n*	%	*n*	%	*n*	%	*n*	%	*n*	%	*n*	%	*n*	%	*n*	%
Gender	Men	710	30.23	31	14.83	104	33.55	75	27.78	80	26.58	93	30.00	92	32.28	133	42.90	50	25.13	52	33.55
	Women	1627	69.26	178	85.17	204	65.81	193	71.48	221	73.42	217	70.00	191	67.02	173	55.81	149	74.87	101	65.16
Place of Residence	Village/Town	1284	54.66	150	71.77	224	72.26	230	85.19	235	78.07	226	72.90	88	30.88	93	30.00	38	19.10	155	100.00
	City	1065	45.34	59	28.23	86	27.74	40	14.81	66	21.93	84	27.10	197	69.12	217	70.00	161	80.90	0	0.00
Level of Study	Bachelor	1843	78.46	143	68.42	226	72.90	137	50.74	171	56.81	291	93.87	245	85.96	283	91.29	196	98.49	151	97.42
	Master	506	21.54	66	31.58	84	27.10	133	49.26	130	43.19	19	6.13	40	14.04	27	8.71	3	1.51	4	2.58
Physical Activity	Sufficient	894	38.06	129	61.72	103	33.23	139	51.48	106	35.22	140	45.16	94	32.98	45	14.52	75	37.69	63	40.65
	Insufficient	1453	61.86	80	38.28	207	66.77	129	47.78	195	64.78	170	54.84	191	67.02	265	85.48	124	62.31	92	59.35
Exposure to COVID-19	Lower	735	31.29	77	36.84	181	58.39	101	37.41	89	29.57	63	20.32	75	26.32	60	19.35	64	32.16	25	16.13
	Higher	1613	68.67	132	63.16	129	41.61	169	62.59	211	70.10	247	79.68	210	73.68	250	80.65	135	67.84	130	83.87
PNIC: Total	Lower	1068	45.47	124	59.33	182	58.71	163	60.37	78	25.91	146	47.10	152	53.33	74	23.87	115	57.79	34	21.94
	Higher	1281	54.53	85	40.67	128	41.29	107	39.63	223	74.09	164	52.90	133	46.67	236	76.13	84	42.21	121	78.06
PNIC: Qualifications	Lower	988	42.06	103	49.28	166	53.55	139	51.48	73	24.25	123	39.68	130	45.61	106	34.19	96	48.24	52	33.55
	Higher	1361	57.94	106	50.72	144	46.45	131	48.52	228	75.75	187	60.32	155	54.39	204	65.81	103	51.76	103	66.45
PNIC: Economic Status	Lower	955	40.66	98	46.89	145	46.77	167	61.85	85	28.24	122	39.35	98	34.39	116	37.42	79	39.70	45	29.03
	Higher	1394	59.34	111	53.11	165	53.23	103	38.15	216	71.76	188	60.65	187	65.61	194	62.58	120	60.30	110	70.97
PNIC: Relationships	Lower	981	41.76	118	56.46	171	55.16	125	46.30	91	30.23	150	48.39	159	55.79	40	12.90	100	50.25	27	17.42
	Higher	1368	58.24	91	43.54	139	44.84	145	53.70	210	69.77	160	51.61	126	44.21	270	87.10	99	49.75	128	82.58
GSRH-1	Better	2120	90.25	190	90.91	295	95.16	228	84.44	279	92.69	299	96.45	234	82.11	264	85.16	190	95.48	141	90.97
	Worst	229	9.75	19	9.09	15	4.84	42	15.56	22	7.31	11	3.55	51	17.89	46	14.84	9	4.52	14	9.03
GSRH-2 Comparative	Better	2030	86.42	181	86.60	278	89.68	213	78.89	260	86.38	297	95.81	220	77.19	269	86.77	178	89.45	134	86.45
	Worst	319	13.58	28	13.40	32	10.32	57	21.11	41	13.62	13	4.19	65	22.81	41	13.23	21	10.55	21	13.55
Life Satisfaction	Lower	927	39.46	76	36.36	84	27.10	52	19.26	150	49.83	124	40.00	123	43.16	223	71.94	67	33.67	28	18.06
	Higher	1422	60.54	133	63.64	226	72.90	218	80.74	151	50.17	186	60.00	162	56.84	87	28.06	132	66.33	127	81.94

*Note*. PNIC = Perceived Negative Impact of Coronavirus on students' well-being; GSRH = General Self-Rated Health.

A required sample size for each country was determined using G*Power software [73]. With a medium effect size *W* = 0.30, an alpha-level of *p* = 0.05, a power of 0.95, and df = 1, a power analysis for the χ^2^ contingency table resulted in a sample size of *n* = 145. To avoid missing data, the survey required participants to complete all answers on each page. Therefore, participants filling in the questionnaire via Google Form did not omit any answers. However, the *hot-deck imputation was introduced to deal with the low number of missing data (n = 5, 0.02%) in the German sample (conducted via SoSci Survey).*

### Statistical Analysis

A preliminary analysis of the proportion of all variables was conducted before statistical tests were applied. First, a one-way ANOVA was performed to test the differences in mean scores of life satisfaction between university students from the following countries: Slovenia, the Czech Republic, Germany, Poland, Ukraine, Russia, Turkey, Israel, and Colombia. The Tukey's honest significant difference (HSD) test was used to find means that were significantly different from each other.

Second, Pearson's χ^2^ independence test was conducted to examine associations between satisfaction with life and other variables. Contingency tables (2 ^x^ 2) were created for each country separately for the following dichotomized variables: satisfaction with life (Lower, Highest), gender (Women, Men), place of residence (Village/Town, City), level of study (Bachelor, Master), level of physical activity (Sufficient, Insufficient), exposure to the COVID-19 pandemic (Lower, Higher), perceived negative impact of COVID-19 on students' well-being, as well as impacts on qualifications, economic status, and social relationships (Lower, Higher), self-rated physical health (Better, Worst), and comparative self-rated physical health (Better, Worst). Students from Colombia (100%) were assigned to the Town/City category, and 97% (*n* = 151) to the first cycle study. Thus, place of residence and level of study were excluded from the statistical analysis of the Colombian sample. The effect size for the χ^2^ test was assessed using the ϕ coefficient.

Next, a logistic regression analysis was performed in each country separately to test the odds ratio (OR) and the adjusted odds ratio (AOR) with 95% *CI*, to find predictors of satisfaction with life. Demographic variables (gender, place of residence, and level of study), physical activity, exposure to COVID-19, perceived negative impact of COVID-19 on students’ well-being, and physical health (general and comparative) were examined as potential predictors of life satisfaction. Statistica 13.3 [74] was performed for statistical analyses.

## Results

### Proportion of satisfaction with life and other variables in the study

The present study found that most university students (60.54%) were satisfied with their life (SWLS > 21). The country with the highest proportion of participants reporting higher SWLS score was Colombia (81.94%) and the lowest was Turkey (28.06%). Most participants had been exposed to COVID-19 (68.67%); the highest rate was in Colombia (83.87%), while the lowest was in the Czech Republic (41.61%). Most participants had a high total PNIC (54.53%), with the highest rate in Colombia (78.06%) and the lowest in Germany (39.63%). The area of qualifications was a leading source of concern for the majority of the student sample (57.94%), with the highest rate of concern occurring in Poland (75.75%) and the lowest in the Czech Republic (46.45%). Additionally, concerns about the PNIC were related to an economic area in 59.34% of university students, with this type of concern being most common in Poland (71.74%) and least common in Germany (38.15%). Social relationships were also perceived to be at risk by most participants (58.24%), with the highest rate of concern among Turkish students and the lowest among Slovenians (43.54%). An in*s*ufficient level of physical activity (PA < 150 min per week) was reported by 61.86% of university students, most frequently in Turkey (85.48%) and less often in Slovenia (38.28%). Few participants complained about their physical health (9.75%), with the lowest rate of complaints occurring among participants from the Ukraine (3.55%) and the highest among those from Russia (17.89%). Worsening health was indicated in 13.58% in the total sample, with the lowest frequency in participants from the Ukraine (4.19%) and the highest in those from Russia (22.81%). The distribution of variables in particular countries is presented in Table 1.

### Differences in satisfaction with life between students from the nine countries

**Fig. 1:**
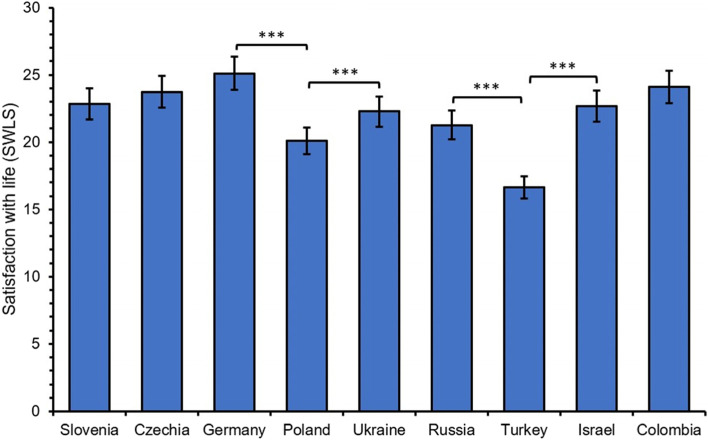
Mean scores of satisfaction with life for university students from the nine countries. Error bars are 95% of confidential interval (*CI*). Differences at the ***p < 0.001 are marked only for the neighboring countries in the figure

Differences between university students from nine countries (Slovenia, Czech Republic, Germany, Poland, Ukraine, Russia, Turkey, Israel, and Colombia) were analyzed using one-way ANOVA. The results indicate that the mean level of life satisfaction significantly differed between participants from different countries with a large effect size (*F*(8, 2340) = 49.81, *p* < 0.001, η_*p*_^2^ = 0.15; see Table 2 and Figure 1 for more details).

**Table 2:** Descriptive statistics regarding satisfaction with life in the nine countries

				95% ***CI***					Cronbach's
Sample	***N***	Range	***M***	***LL***	***UL***	***SD***	Skewness	Kurtosis	α
Total	2349	5-35	21.85	21.58	22.11	6.54	-0.36	-0.49	0.85
Slovenia	209	7-35	22.85	21.97	23.72	6.40	-0.30	-0.53	0.86
Czechia	310	8-35	23.73	23.06	24.39	5.99	-0.45	-0.50	0.84
Germany	270	9-35	25.11	24.46	25.76	5.43	-0.70	0.14	0.83
Poland	301	5-33	20.09	19.36	20.82	6.44	-0.20	-0.57	0.87
Ukraine	310	6-35	22.27	21.61	22.93	5.92	-0.06	-0.46	0.83
Russia	285	5-35	21.26	20.54	21.98	6.18	-0.17	-0.19	0.84
Turkey	310	5-32	16.64	15.87	17.41	6.89	0.29	-0.78	0.86
Israel	199	7-33	22.67	21.91	23.43	5.45	-0.53	-0.21	0.84
Colombia	155	9-34	24.10	23.33	24.86	4.84	-0.79	0.65	0.60

*Note*. *M* = mean; *CI* = confidence interval; *LL* – lower limit; *UL* = upper limit; *SD* – standard deviation.

University students from Slovenia had significantly higher scores in the SWLS than their counterparts from Poland (*p* < 0.001) and Turkey (*p* < 0.001), while they had lower scores than those from Germany (*p* < 0.01). Satisfaction with life was higher among individuals from the Czech Republic than among those from Poland (*p* < 0.001), Russia (*p* < 0.001), and Turkey (*p* < 0.001). University students from Germany presented a higher level of life satisfaction than students from other countries. Statistically significant differences were found in comparison to Poland (*p* < 0.001), Slovenia (*p* < 0.01), Ukraine (*p* < 0.001), Russia (*p* < 0.001), Turkey (*p* < 0.001), and Israel (*p* < 0.001). Students from Poland had significantly higher scores in the SWLS than participants from Turkey (*p* < 0.001) but lower than those from Slovenia, the Czech Republic (*p* < 0.001), Germany (*p* < 0.001), Ukraine (*p* < 0.001), and Israel (*p* < 0.001). The level of life satisfaction of Ukrainian students was significantly higher than that of their counterparts from Turkey (*p* < 0.001) and lower than that of participants from Germany (*p* < 0.001). Russian students showed significantly lower life satisfaction than participants from the Czech Republic (*p* < 0.001), Germany (*p* < 0.001) and Colombia (*p* < 0.001), while they had higher scores than individuals from Turkey (*p* < 0.001). Turkish students had the lowest scores in the SWLS, and significant differences (*p* < 0.001) were found in comparison to all other eight countries. Participants from Israel showed a higher level of satisfaction with life than students from Poland (*p* < 0.001) and Turkey (*p* < 0.001) and a lower level of satisfaction than students from Germany (*p* < 0.001). Life satisfaction in Colombian students was higher than in their counterparts from Poland (*p* < 0.001), Russia (*p* < 0.001), and Turkey (*p* < 0.001).

### Associations between satisfaction with life and other variables

Associations between life satisfaction and other variables were determined using Pearson's χ^2^ independence test to assess the nine countries (details are included in Table 3). Gender was associated with satisfaction with life in Germany and Russia. Higher life satisfaction was reported more frequently among females in Germany (*p* < 0.01, ϕ = 0.16) and males in Russia (*p* < 0.001, ϕ = -0.21). A slight predominance of individuals with high life satisfaction was found for Polish (*p* < 0.05, ϕ = 0.13) and Israeli (*p* < 0.05, ϕ = 0.14) university students living in the city, when compared with those from villages or towns. Higher life satisfaction was more likely among students studying at a masters level than compared with those studying at a bachelors level in the Czech Republic (*p* < 0.01, ϕ = 0.18) and Poland (*p* < 0.01, ϕ = 0.16). A sufficient level of physical activity was slightly more prevalent than an insufficient level among participants with higher levels of satisfaction with life in Slovenia (*p* < 0.05, ϕ = 0.14) and Ukraine (*p* < 0.05, ϕ = 0.12).

**Table 3:** Association of satisfaction with life and other variables in the nine countries

	Satisfaction with Life																	
	Slovenia (*n* = 209)		Czechia (*n* = 308)		Germany (*n* = 267)		Poland (*n* = 301)		Ukraine (*n* = 310)		Russia (*n* = 285)		Turkey (*n* = 310)		Israel (*n* = 199)		Colombia (*n* = 153)	
	Lower	Higher	Lower	Higher	Lower	Higher	Lower	Higher	Lower	Higher	Lower	Higher	Lower	Higher	Lower	Higher	Lower	Higher
Variable	n(%)	n(%)	n(%)	n(%)	n(%)	n(%)	n(%)	n(%)	n(%)	n(%)	n(%)	n(%)	n(%)	n(%)	n(%)	n(%)	n(%)	n(%)
Gender	χ^2^(1) = 0.85		χ^2^(1) = 1.82		χ^2^(1) = 7.17**		χ^2^(1) = 0.67		χ^2^(1) = 1.13		χ^2^(1) = 12.25***		χ^2^(1) = 0.07		χ^2^(1) = 0.00		χ^2^(1) = 0.05	
Men	9(29)	22(71)	33(32)	71(68)	22(29)	53(71)	43(54)	37(46)	33(35)	60(65)	26(28)	66(72)	95(71)	38(29)	17(34)	33(66)	9(17)	43(83)
Women	67(38)	111(62)	50(25)	154(75)	29(15)	164(85)	107(48)	114(52)	91(42)	126(58)	96(50)	95(50)	126(73)	47(27)	50(34)	99(66)	19(19)	82(81)
Place of Residence	χ^2^(1) = 0.61		χ^2^(1) = 1.11		χ^2^(1) = 1.38		χ^2^(1) = 4.83*		χ^2^(1) = 2.13		χ^2^(1) = 3.30		χ^2^(1) = 0.09		χ^2^(1) = 3.95*			
Village/Town	57(38)	93(62)	57(25)	167(75)	47(20)	183(80)	125(53)	110(47)	96(42)	130(58)	45(51)	43(49)	68(73)	25(27)	18(47)	20(53)		
City	19(32)	40(68)	27(31)	59(69)	5(12)	35(88)	25(37)	41(62)	28(33)	56(67)	78(40)	119(60)	155(71)	62(29)	49(30)	112(70)		
Level of Study	χ^2^(1) = 0.38		χ^2^(1) = 9.57**		χ^2^(1) = 1.25		χ^2^(1) = 7.52**		χ^2^(1) = 0.60		χ^2^(1) = 0.01		χ^2^(1) = 1.33		χ^2^(1) = 0.00			
Bachelor	54(38)	89(62)	72(32)	154(68)	30(22)	107(78)	97(57)	74(43)	118(41)	173(59)	106(43)	139(57)	201(71)	82(29)	66(34)	130(66)		
Master	22(33)	44(67)	12(14)	72(86)	22(17)	111(83)	53(41)	77(59)	6(32)	13(68)	17(43)	23(58)	22(81)	5(19)	1(33)	2(67)		
Physical Activity	χ^2^(1) = 4.18*		χ^2^(1) = 3.51		χ^2^(1) = 1.56		χ^2^(1) = 2.71		χ^2^(1) = 4.40*		χ^2^(1) = 0.03		χ^2^(1) = 1.46		χ^2^(1) = 0.49		χ^2^(1) = 3.48	
Sufficient	40(31)	89(69)	21(20)	82(80)	23(17)	116(83)	46(43)	60(57)	47(34)	93(66)	40(43)	54(57)	29(64)	16(36)	23(31)	52(69)	7(11)	56(89)
Insufficient	36(45)	44(55)	63(30)	144(70)	28(22)	101(78)	104(53)	91(47)	77(45)	93(55)	83(43)	108(57)	194(73)	71(27)	44(35)	80(65)	21(23)	71(77)
Exposure to COVID	χ^2^(1) = 17.42***		χ^2^(1) = 11.44***		χ^2^(1) = 5.65*		χ^2^(1) = 3.59		χ^2^(1) = 1.46		χ^2^(1) = 15.23***		χ^2^(1) = 10.57**		χ^2^(1) = 0.67		χ^2^(1) = 2.04	
Lower	14(18)	63(82)	36(20)	145(80)	12(12)	89(88)	37(42)	52(58)	21(33)	42(67)	18(24)	57(76)	33(55)	27(45)	19(30)	45(70)	2(8)	23(92)
Higher	62(47)	70(53)	48(37)	81(63)	40(24)	129(76)	113(54)	98(46)	103(42)	144(58)	105(50)	105(50)	190(76)	60(24)	48(36)	87(64)	26(20)	104(80)
PNIC: Total	χ^2^(1) = 10.54**		χ^2^(1) = 0.74		χ^2^(1) = 15.29***		χ^2^(1) = 5.45*		χ^2^(1) = 1.57		χ^2^(1) = 1.80		χ^2^(1) = 7.50**		χ^2^(1) = 4.16*		χ^2^(1) = 1.17	
Lower	34(27)	90(73)	46(25)	136(75)	19(12)	144(88)	30(38)	48(62)	53(36)	93(64)	60(39)	92(61)	44(59)	30(41)	32(28)	83(72)	4(12)	30(88)
Higher	42(49)	43(51)	38(30)	90(70)	33(31)	74(69)	120(54)	103(46)	71(43)	93(57)	63(47)	70(53)	179(76)	57(24)	35(42)	49(58)	24(20)	97(80)
PNIC: Qualifications	χ^2^(1) = 7.39**		χ^2^(1) = 0.06		χ^2^(1) = 15.55***		χ^2^(1) = 7.79**		χ^2^(1) = 3.76		χ^2^(1) = 0.26		χ^2^(1) = 1.96		χ^2^(1) = 2.55		χ^2^(1) = 0.50	
Lower	28(27)	75(73)	46(28)	120(72)	14(10)	125(90)	26(36)	47(64)	41(33)	82(67)	54(42)	76(86)	71(67)	35(33)	27(28)	69(72)	11(21)	41(79)
Higher	48(45)	58(55)	38(26)	106(74)	38(29)	93(71)	124(54)	104(46)	83(44)	104(56)	69(45)	86(55)	152(75)	52(25)	40(39)	63(61)	17(17)	86(83)
PNIC: ES	χ^2^(1) = 11.24***		χ^2^(1) = 1.21		χ^2^(1) = 0.47		χ^2^(1) = 8.46**		χ^2^(1) = 0.81		χ^2^(1) = 4.36*		χ^2^(1) = 4.87*		χ^2^(1) = 0.24		χ^2^(1) = 3.61	
Lower	24(24)	74(76)	35(24)	110(76)	30(18)	137(82)	31(36)	54(64)	45(37)	77(63)	34(35)	64(65)	75(65)	41(35)	25(32)	54(68)	4(9)	41(91)
Higher	52(47)	59(53)	49(30)	116(70)	22(21)	81(79)	119(55)	97(45)	79(42)	109(58)	89(48)	98(52)	148(76)	46(24)	42(35)	78(65)	24(22)	86(78)
PNIC: Relationships	χ^2^(1) = 5.26*		χ^2^(1) = 5.75*		χ^2^(1) = 1.59		χ^2^(1) = 6.75**		χ^2^(1) = 23.44		χ^2^(1) = 1.24		χ^2^(1) = 16.50***		χ^2^(1) = 0.24		χ^2^(1) = 1.07	
Lower	35(30)	83(70)	37(22)	134(78)	20(16)	105(84)	35(38)	56(62)	52(35)	98(65)	64(40)	95(60)	18(45)	22(55)	32(32)	68(68)	3(11)	24(89)
Higher	41(45)	50(55)	47(34)	92(66)	32(22)	113(78)	115(55)	95(45)	72(45)	88(55)	59(47)	67(53)	205(76)	65(24)	35(35)	64(65)	25(20)	103(80)
GSRH-1 (Low)	χ^2^(1) = 12.58***		χ^2^(1) = 5.49*		χ^2^(1) = 14.40***		χ^2^(1) = 12.70***		χ^2^(1) = 5.09*		χ^2^(1) = 6.21*		χ^2^(1) = 6.04*		χ^2^(1) = 12.87***		χ^2^(1) = 6.39*	
Better	62(33)	128(67)	76(26)	219(74)	35(15)	193(85)	131(47)	148(53)	116(39)	183(61)	93(40)	141(60)	183(69)	81(31)	59(31)	131(69)	22(16)	119(84)
Worst	14(74)	5(26)	8(53)	7(47)	17(40)	25(60)	19(86)	3(14)	8(73)	3(27)	30(59)	21(41)	40(87)	6(13)	8(89)	1(11)	6(43)	8(57)
GSRH-2 Comparative	χ^2^(1) = 6.03*		χ^2^(1) = 15.35***		χ^2^(1) = 5.19*		χ^2^(1) = 15.11***		χ^2^(1) = 1.08		χ^2^(1) = 11.60***		χ^2^(1) = 7.85**		χ^2^(1) = 5.79*		χ^2^(1) = 3.82	
Better	60(33)	121(67)	66(24)	212(76)	35(16)	178(84)	118(45)	142(55)	117(39)	180(61)	83(38)	137(62)	186(69)	83(31)	55(31)	123(69)	21(16)	113(84)
Worst	16(57)	12(43)	18(56)	14(44)	17(30)	40(70)	32(78)	9(22)	7(54)	6(46)	40(62)	25(38)	37(90)	4(10)	12(57)	9(43)	7(33)	14(67)

*Note*. PNIC = Perceived Negative Impact of Coronavirus on students’ well-being; ES = Economic Status; GSRH = General Self-Rated Health. *p < 0.05, **p < 0.01, ***p < 0.001.

Significantly more respondents with higher levels of life satisfaction reported low exposure to COVID-19 in Slovenia (*p* < 0.001, ϕ = -0.29), the Czech Republic (*p* < 0.001, ϕ = -0.19), Russia (*p* < 0.001, ϕ = -0.23), Turkey (*p* < 0.01, ϕ = -0.18), and Germany (*p* < 0.05, ϕ = -0.15). The perceived negative impact of coronavirus on students’ well-being (PNIC) was not associated with life satisfaction in Ukraine or Colombia. In Slovenia and Poland, students with higher levels of life satisfaction were more likely to report lower PNIC scores (*p* < 0.01, ϕ = -0.23, and *p* < 0.05, ϕ = -0.14, respectively) as well as lower scores regarding PNIC qualifications (*p* < 0.01, ϕ = -0.19, and *p* < 0.01, ϕ = -0.16, respectively), economic status (*p* < 0.001, ϕ = -0.23, and *p* < 0.01, ϕ = -0.17, respectively), and social relationships (*p* < 0.05, ϕ = -0.16, and *p* < 0.01, ϕ = -0.15, respectively). Among Turkish university students, those with higher scores regarding satisfaction with life more frequently had lower PNIC scores in terms of both social relationships (*p* < 0.001, ϕ = -0.23) and economics (*p* < 0.05, ϕ = -0.13), as well as lower total scores (*p* < 0.01, ϕ = -0.16). In Germany, significantly more participants with higher levels of life satisfaction had lower total PNIC scores (*p* < 0.001, ϕ = -0.24) as well as lower scores regarding anxiety about qualifications (*p* < 0.001, ϕ = -0.24) when compared with their counterparts, who reported higher PNIC scores. In the sample of Russian students who were more satisfied with their lives, a lower economic status PNIC was reported more frequently (*p* < 0.05, ϕ = -0.12). Additionally, people with lower total PNIC scores were slightly more prevalent in the Israeli population (*p* < 0.05, ϕ = -0.15).

A better GSRH-1 was associated with higher life satisfaction in all countries: Slovenia (*p* < 0.001, ϕ =- 0.25), the Czech Republic (*p* < 0.05, ϕ = -0.13), Germany (*p* < 0.001, ϕ =- 0.23), Poland (*p* < 0.001, ϕ = -0.21), Ukraine (*p* < 0.05, ϕ = -0.13), Russia (*p* < 0.05, ϕ = -0.15), Turkey (*p* < 0.05, ϕ = -0.14), Israel (*p* < 0.001, ϕ = -0,25.), and Colombia (*p* < 0.05, ϕ = -0.20). Additionally, a better comparative GSRH-2 was related to higher satisfaction with life in most countries (beside Ukraine and Colombia): Slovenia (*p* < 0.05, ϕ = -0.17), Czech Republic (*p* < 0.001, ϕ = -0.22), Germany (*p* < 0.05, ϕ = -0.14), Poland (*p* < 0.001, ϕ = -0.22), Russia (*p* < 0.001, ϕ = -0.20), Turkey (*p* < 0.01, ϕ = -0.16), and Israel (*p* < 0.05, ϕ = -0.17).

### Predictors of satisfaction with life

A logistic regression was performed to identify significant predictors of life satisfaction among a set of variables that were previously included in the relationship analysis using the Pearson χ^2^ test (see Table 4 for more details). Females from Germany were 2-3 times more likely to rate their life satisfaction highly than males (OR = 3.35, 95% *CI* = 1.24, 4.44; AOR = 3.24, 95% *CI* = 1.55, 6.77). However, half as many Russian males than females were satisfied with their lives. (OR = 0.39, 95% *CI* = 0.23, 0.67; AOR = 0.35, 95% *CI* = 0.19, 0.63). The multivariate model showed that about twice as many Russian (AOR = 2.38, 95% *CI* = 1.03, 3.13) and Israeli (AOR =1.79, 95% *CI* = 1.10, 5.17) university students who were satisfied with their lives were living in a city than in a village or town, but no effect was identified in a bivariate regression model. Half as many Polish students living in the city considered their lives to be satisfying compared with those from a village or town, but this effect was only statistically significant in the bivariate model (OR = 0.54, 95% *CI* = 0.34, 0.94). Individuals from the Czech Republic (OR = 2.81, 95% *CI* = 1.43, 5.51; AOR = 2.94, 95% *CI* = 1.39, 6.20) studying at the masters level were three times more likely Masters students from Poland (OR = 0.53, 95% *CI* = 0.33, 0.84; AOR = 0.49, 95% *CI* = 0.29, 0.82) were two times more likely to have high life satisfaction when compared with their counterparts at the bachelor degree. The bivariate regression showed that having a sufficient level of PA is a predictor of satisfaction with life for participants from Slovenia (OR = 0.55, 95% *CI* = [0.31, 0.98]) and Ukraine (OR = 1.64, 95% *CI* = 1.03, 2.61), and this was also shown for those from the Czech Republic (AOR = 1.91, 95% *CI* = 1.03, 3.56) in a multivariate model of logistic regression.

**Table 4:** Logistic regression for satisfaction with life in the nine countries

	Slovenia (*n* = 209)		Czechia (*n* = 308)		Germany (*n* = 267)		Poland (*n* = 301)		Ukraine (*n* = 310)		Russia (*n* = 285)		Turkey (*n* = 310)		Israel (*n* = 199)		Colombia (*n* = 153)	
Variable	OR	AOR	OR	AOR	OR	AOR	OR	AOR	OR	AOR	OR	AOR	OR	AOR	OR	AOR	OR	AOR
Gender	1.48	1.11	1.43	1.36	2.35**	3.24**	0.81	0.74	0.76	0.87	0.39**	0.35***	0.93	0.92	1.02	1.01	1.11	1.25
Place of Residence	0.78	0.71	0.75	0.73	1.80	2.34	0.54*	0.57	1.48	1.42	1.60	1.79*	1.09	0.80	2.06	2.38*		
Level of Study	0.82	1.13	2.81**	2.94**	1.41	1.18	0.53**	0.49**	1.48	1.22	1.03	1.12	0.56	0.48	1.02	2.23		
PA	0.55*	0.56	1.71	1.91*	1.40	0.96	0.67	0.81	1.64*	1.56	1.04	0.89	1.51	1.44	1.24	1.05	0.42	0.50
Exposure to COVID-19	3.99***	2.63*	0.42***	0.42**	0.43*	0.44*	1.62	1.28	0.70	0.81	0.53*	0.54*	0.39**	0.42**	0.77	0.89	2.88	3.21
PNIC: Total	2.59***	0.90	0.80	1.61	0.30***	0.37	1.86*	0.44	0.75	1.55	0.72	1.19	0.47**	1.10	0.54*	0.49	1.85	1.45
PNIC: Qualifications	2.22**	1.36	1.07	1.43	0.27***	0.46	2.16**	1.96	0.63	0.58	0.89	1.01	0.69	0.94	0.62	0.89	0.74	0.28*
PNIC: Economic Status	2.72***	2.09	0.75	0.67	0.81	1.66	2.14**	2.68**	0.81	0.86	0.58*	0.81	0.57*	0.70	0.86	1.33	2.86	2.92
PNIC: Relationships	1.94*	1.63	0.54*	0.53	0.67	1.24	1.94**	1.89	0.65	0.56	0.77	0.73	0.47**	0.24***	0.86	1.30	1.94	1.72
GSRH-1	5.78***	4.12*	0.30*	0.88	0.27***	0.36*	7.16**	4.21	0.24*	0.25	0.46*	0.69	0.34*	0.61	0.06**	0.06*	4.06*	3.06
GSRH-2 Comparative	2.69*	1.23	0.24***	0.34*	0.46*	0.76	4.28***	2.89*	0.30	0.99	0.38***	0.39*	0.24**	0.50	0.34*	0.81	2.69	1.51

*Note*. PA = Physical Activity; PNIC = Perceived Negative Impact of Coronavirus on students’ well-being; GSRH = General Self-Rated Health; OR = Odds Ratio; AOR = Adjusted Odds Ratio. *p < 0.05, **p < 0.01, ***p < 0.001

Both the bivariate and multivariate models showed that participants from Slovenia who had not been exposed to COVID-19 were 2.5-4 times more likely (OR = 3.99, 95% *CI* = 2.03, 7.8]; AOR = 2.63, 95% *CI* = 1.25, 5.56) and participants from the Czech Republic (OR = 0.42, 95% *CI* = 0.25, 0.70; AOR = 0.42, 95% *CI* = 0.24, 0.74), Germany (OR = 0.43, 95% *CI* = 0.22, 0.88; AOR = 0.44, 95% *CI* = 0.20, 0.98), Russia (OR = 0.53, 95% *CI* = 0.31, 0.90; AOR = 0.54, 95% *CI* = 0.31, 0.93), and Turkey (OR = 0.39, 95% *CI* = 0.22, 0.70; AOR = 0.42, 95% *CI* = 0.22, 0.80) were about 2 times more likely to report a high level of life satisfaction (when compared with those who had experienced greater exposure to coronavirus).

The low total perceived negative impact of coronavirus (PNIC) on the daily lives of students was identified as a significant predictor of higher life satisfaction among students from Slovenia (OR = 2.59, 95% *CI* = 1.44, 4.63), Germany (OR = 0.30, 95% *CI* = 0.16, 0.56), Poland (OR = 1.86, 95% *CI* = 1.10, 3.16), Turkey (OR = 0.47, 95% *CI* = 0.27, 0.81), and Israel (OR = 0.54, 95% *CI* = 0.30, 0.98), but only in a bivariate model of regression. Students from Slovenia (OR = 2.22, 95% *CI* = 1.24, 3.97), Germany (OR = 0.27, 95% *CI* = 0.14, 0.54), Poland (OR = 2.16, 95% *CI* = 1.25, 3.73), and Colombia (AOR = 0.28, 95% *CI* = 0.09, 0.90) with a lower PNIC in the area of Qualifications were significantly more satisfied with their lives. Individuals from Slovenia (OR = 2.72, 95% *CI* = 1.50, 4.93), Poland (OR = 2.14, 95% *CI* = 1.27, 3.59; AOR = 2.68, 95% *CI* = 1.42, 5.06), Russia (OR = 0.58, 95% *CI* = 0.35, 0.97), and Turkey (OR = 0.57, 95% *CI* = 0.34, 0.94) who were less worried about their economic status were more satisfied with their lives. The lower the perceived negative impact of COVID-19 on social relationships, the higher the level of life satisfaction noted among university students from Slovenia (OR = 1.94, 95% *CI* = 0.02, 1.94), the Czech Republic (OR = 0.54, 95% *CI* = 0.33, 0.90), Poland (OR = 1.94, 95% *CI* = 1.17, 3.21), and Turkey (OR = 0.26, 95% *CI* = 0.13, 0.51; AOR = 0.24, 95% *CI* = 0.10, 0.58).

In the bivariate regression model, good physical health was indicated 2-7 times more frequently among participants with a high level of life satisfaction when compared with those with the worst health status in all countries of interest: Slovenia (OR = 5.78, 95% *CI* = 1.98, 16.88), the Czech Republic (OR = 0.30, 95% *CI* = 0.11, 0.87), Germany (OR = 0.27, 95% *CI* = 0.13, 0.55), Poland (OR = 7.16, 95% *CI* = 2.06, 24.84), Ukraine (OR = 0.24, 95% *CI* = 0.06, 0.92), Russia (OR = 0.46, 95% *CI* = 0.25, 0.86), Turkey (OR = 0.34, 95% *CI* = 0.14, 0.83), Israel (OR = 0.06, 95% *CI* = 0.01, 0.47), and Colombia (OR = 4.06, 95% *CI* = 1.27, 13.96). However, in the multivariate model, this pattern was only presented in Slovenia (AOR = 4.12, 95% *CI* = 1.11, 15.31), Germany (AOR = 0.36, 95% *CI* = 0.13, 0.98), and Israel (AOR = 0.06, 95% *CI* = 0.01, 0.70). Additionally, having a better comparative GSRH-2 score was a predictor of satisfaction with life for students from Slovenia (OR = 2.69, 95% *CI* = 1.19, 6.07), the Czech Republic (OR = 0.24, 95% *CI* = 0.11, 0.51; AOR = 0.34, 95% *CI* = 0.14, 0.83), Germany (OR = 0.46, 95% *CI* = 10.24, 0.91), Poland (OR = 4.28, 95% *CI* = 1.96, 9.35; AOR = 2.89, 95% *CI* = 1.14, 7.34), Russia (OR = 0.38, 95% *CI* = 0.21, 0.67; AOR = 0.39, 95% *CI* = 0.19, 0.81), Turkey (OR = 0.24, 95% *CI* = 0.08, 0.70), and Israel (OR = 0.34, 95% *CI* = 0.13, 0.85). Comparative GSRH-2 was not found to be a predictor of life satisfaction for students from Ukraine and Colombia.

## Discussion

This study explored cross-national differences in life satisfaction and its predictors during the COVID-19 pandemic among university students from nine countries. Successful adaptation to life regarding school, work, and family is related to a high level of life satisfaction [1–3]. Liu et al. [56] showed that the psychological well-being of undergraduate students could influence their academic and professional success and the development of society as a whole. Therefore, studies of student life satisfaction at the beginning of adult life and the related challenges seem necessary, especially in relation to the COVID-19 pandemic.

Our results suggest that there are differences between countries in terms of the number of university students with high and low scores, as well as in the average level of life satisfaction and associations between satisfaction with life and other variables. The highest mean level of life satisfaction was reported among university students from Germany (*M* = 25.11, which means that they are "slightly satisfied with their life"), and the lowest was reported among their counterparts from Turkey (*M* = 16.64, which is interpreted as "slightly dissatisfied"). The low level of life satisfaction of Turkish students may be related to the current volatile economic situation [75] and the high unemployment rate [76]. Substantial individual differences in life satisfaction as well as between cultures have been shown in numerous previous studies [2]. Diener et al. [2] explained these differences as being related to genetic individual differences that predispose people to a more or less positive state as well as to cross-cultural norms regarding positive emotions. At the cross-cultural level, satisfaction with life depends on some ubiquitous needs, such as the need for temperature control, food, income, health, environmental control, and social relationships. These needs can drive people's desires, be used to set goals to be achieved, and provide a level of comparison with others, ultimately driving the subjective assessment of life satisfaction.

Although most university students from the nine countries were satisfied with their lives (61%), the highest rate of satisfaction occurred for students from Colombia (82%) and the lowest was for students from Turkey (28%). Decreases in well-being due to COVID-19 have previously been shown in adult populations from many countries [25, 29–39] as well as in university and college students [39, 42–45, 56–63]. The present study found large differences between student samples from different countries, indicating a high level of disparity in self-rated life satisfaction. It may be dependent on culture rather than on the current state and perceived risk of the COVID-19 pandemic, since students from Colombia were the most likely to report a high level of life satisfaction but also experienced the highest rate of exposure to coronavirus (84%) and had the greatest PNIC scores (78%) in comparison to their counterparts from the other eight countries.

Exposure to COVID-19 may explain, to some degree, a low level of satisfaction with life among participants, but not in all nine countries. Overall, the majority of university students stated that they had experienced some level of exposure to COVID-19, including symptoms of coronavirus infection (and related testing, isolation from loved ones, strict quarantine for at least 14 days, hospitalization) recognized in themselves or in family members or friends or losing a job or experiencing a worsening economic status because of the coronavirus. Moreover, individuals who reported exposure to the coronavirus were more likely to demonstrate a low level of life satisfaction during the first wave of the pandemic. The present study suggests that university students from Slovenia who had not been exposed to COVID-19 were three times more likely and those from the Czech Republic, Germany, Russia, and Turkey were two times more likely to indicate a high level of life satisfaction than people experiencing the effects of the pandemic. Previous research showed that exposure to COVID-19 and a perceived negative impact of the pandemic on livelihood are predictors of mental health problems [77].

Most university students indicated a perceived negative impact of coronavirus (PNIC) on their well-being (54%). The primary sources of coronavirus-related concerns among students were their economic status (59%), social relationships (58%), and qualifications (58%). The perceived impacts of these variables on well-being were noted by 20-40% of participants, depending on the country (there were less cross-national differences regarding social relationships and more regarding qualifications). The level of disparity may be related to the degree of changes in health care politics due to the COVID-19 pandemic, the degree of distancing, and the better or worse organization of the transition process from classroom learning to online e-learning.

This study found an association between high life satisfaction and a low perceived negative impact of coronavirus (PNIC) in most countries but not in Ukraine and Colombia. However, this association was weak and was present only in the bivariate model of regression. Previous research indicates that subjectively assumed restrictions during lockdown were associated with poor mental health in a large nationwide community sample of adults from Germany [78]. In particular, a more significant perceived change in life related to a reduction in social contact caused higher impairments in areas of well-being such as loneliness, psychosocial distress, anxiety and depression symptoms, fear, and life-satisfaction [31, 34, 35, 54, 78, 79]. Gawrych et al. [31] showed that decreases in the level of happiness and life satisfaction during the first stage of the COVID-19 pandemic in Poland were associated with anxiety about the COVID-19 pandemic, including concerns about loved ones, healthcare failure, and consequences of the pandemic at individual and social levels.

The present study found that anxiety about getting the right qualifications was a significant predictor of lower life satisfaction among students from Slovenia, Germany, Poland, and Colombia. Competence and capability seem to be essential for the well-being of university students. A previous study [80] showed that perceived teaching competencies might positively predict school satisfaction and satisfaction with life among physical education students. Unfortunately, the transformation of the education system during lockdown was very stressful due to internet connection problems and the significant amount of time required to master new technologies, including software and hardware [20–23]. Research exploring the impact of the pandemic on the students' well-being found that the coronavirus outbreak affected the amount of time spent doing classes and self-study online. This affected learning, sleeping habits, and the daily fitness routine and had subsequent effects on weight, social life, and mental health [21]. Additionally, university teachers experienced high levels of emotional exhaustion, mental overload, and time pressure, as well as stress due to lacking a schedule during the rapid transition process from classroom to e-learning [22]. This may have translated into a sense of stress among students and a reduction in the quality of education.

Participants from Slovenia, Poland, Russia, and Turkey who did not notice a drop in income were more satisfied with their lives. This result is consistent with previous research that showed a positive association between having a high income and life satisfaction both before [2] and during the COVID-19 pandemic [77]. In particular, job and income loss during the coronavirus outbreak caused a deep decline in life satisfaction in the UK male population [13].

Disruption in social relationships, as a leading source of concerns, was identified as a predictor of lower life satisfaction in students from Slovenia, Czechia, Poland, and Turkey. Social support is an important factor that affects people's lives and well-being. Previous findings suggest that undergraduates with higher social support levels have substantially greater adaptability and life satisfaction than university students with lower social support levels [17]. Another study showed that perceived social isolation is associated with low life satisfaction [30]. A research conducted before the COVID-19 outbreak indicated that perceived social support and resilience play mediating roles as protective buffers in the relationship between perceived stress and life satisfaction [16]. On the other hand, a recent study found that psychological and social distancing can play mediation roles and explain how the COVID-19 pandemic impacts both perceived general health and life satisfaction [81]. This study seems to confirm that social isolation is an essential factor in student's well-being.

Most of the university students studied self-rated their physical health as good (almost 90%). However, participants who had experienced a worse health status were 2-7 times more likely to be unhappy than people with good health. This pattern of bivariate association was significant in all nine countries. When the other variables were included in the multivariate regression model, the association was shown to be much weaker and only occurred in Slovenia, Germany, and Israel. Apart from in participants from Ukraine and Colombia, comparatively better self-rated health was also a predictor of higher satisfaction with life. The strongest relationships were noted in Poland and Slovenia, with those with comparatively good levels of health being 4 and 2.5 times more satisfied than those with poor health, respectively. The positive association between health and satisfaction with life was also shown in previous studies (e.g.,82-84) and is consistent with Diener's concept of satisfaction with life [1, 2].

The study identified a positive association between engaging in a sufficient level of PA and life satisfaction level for university students from Slovenia, Ukraine, and the Czech Republic. However, the effect size was weak. This result is consistent with previous studies performed during the COVID-19 pandemic that showed that PA has a beneficial effect on mental health by reducing nervous tension and negative emotions, decreasing depression and anxiety, and improving sleep quality and well-being [38, 39, 42, 43, 49–52]. A low level of PA and prolonged sitting time during lockdown were associated with a poor health-related quality of life and a high level of perceived stress among Chinese adults [41]. PA can also be seen as a protective factor against the risk of coronavirus infection [52, 85]. Therefore, the low level of PA among university students found in this study is alarming. Most participants (62%) reported an in*s*ufficient level of PA, where “sufficient” was defined as a minimum of 150 minutes per week, as recommended by the WHO. The number of physically active students differed between countries, ranging from high in Slovenia (50%, the highest frequency of active students) to low in Turkey (the lowest number of people with a sufficient PA level). The level of disparity between countries may be related to cross-cultural differences. The low levels of PA among university students found in this study may be due to coronavirus-related restrictions and the recommendation to stay at home. Exercising outside the home was prohibited during the lockdown period, and sports and fitness clubs were closed in most of the countries studied. Previous studies indicate that the level of PA decreased during the COVID-19 pandemic in populations from many countries worldwide [38, 47, 86, 87].

The study did not find an association between gender and life satisfaction, apart from weak associations in Germany (females more satisfied) and Russia (males more satisfied). Previous research found a negative relationship between mental health and well-being and female gender during the COVID-19 pandemic [39–48]. Additionally, male participants from Spain were found to be more satisfied with their lives than females in a previous study [54]. Research conducted in Israel found that females have a high risk for developing secondary effects from the COVID-19 pandemic such as decreased well-being in both psychological and social life areas [34]. On the other hand, a recent study performed globally in 166 countries found small gender differences with higher levels of life satisfaction in females than males [88]. A higher rate of satisfaction with life among females than males was found in German students, which seems to confirm these general trends. The COVID-19 pandemic may have had a less disruptive effect in such a stable country as Germany, whereas in Russia, changes related to lockdown caused decreasing life satisfaction in females. Bambra et al. [40] suggested that gender-based health inequalities arising from the COVID-19 pandemic have led to an increased level mortality among males and an increased level of morbidity among females. This may explain why gender differences changed during the COVID-19 pandemic, and decreased life satisfaction was more common in females (compared with males). More research is necessary to examine the association between gender and life satisfaction during the COVID-19 pandemic.

This study found a positive association between satisfaction with life and living in the city (in contrast to the countryside) among students from Poland, Russia, and Israel. Previous research indicated that the prevalence of COVID-19 infection in rural areas was substantially higher than in urban areas, especially among obese people and smokers [89]. However, rural areas tend to have a more limited ability to adapt to coronavirus challenges in terms of hospital resources, internet connectivity, satisfactory employment, and income compared with urban areas. This higher level of uncertainty may decrease life satisfaction among students living in the countryside. Additionally, participants from the Czech Republic and Poland, studying at the master level, were more likely to assess their life as satisfying when compared with their counterparts studying at the bachelor level. This result is consistent with a previous study conducted among undergraduates on the relationship between study year and symptoms of mental health disorder [56]. The highest levels of stress, anxiety, and depression were found in first and second year students on average, but some improvements in well-being were achieved in the third and last years of study. This suggests that along with the duration of their studies, students improve their ability to adapt to the university's conditions and develop more efficient coping strategies.

### Limitation of the study

Although this study identified significant predictors of life satisfaction in a large sample of university students from nine countries, the findings should be interpreted with caution due to the cross-sectional design. We cannot determine whether potential predictors preceded the current level of satisfaction with life. Therefore, the causal relationships identified may be biased. Longitudinal research is required in the future to verify the findings of the current study. The online method of recruiting participants to gain a convenience sample using social media and e-mailing a list of students does not allow us to generalize the results of this study to the population of university students as a whole. Self-reported measures may also be a source of bias. Furthermore, we have used a relatively new set of measurements: Exposure to COVID-19, PNIC, and PA [43] which has not yet been well-validated.

## Conclusions

This study identified a significant association between satisfaction with life and various demographic and health-related variables in university students from nine countries. The results of this study could be used as preliminary evidence of causal relationship between life satisfaction and exposure to COVID-19, the perceived negative impact of COVID-19 on students' well-being, physical health, physical activity, and some demographic risk factors (like gender, place of residence, and study level). The relationship of life satisfaction with most variables of interest seems to be related to cross-cultural differences, since a wide range of cross-national differences were found in terms of both prevalence rates and relationships between variables. However, an association between life satisfaction and subjective physical health assessment suggests that there is a universal pattern that is relatively independent of country. Therefore, each country should develop individual strategies to cope with the COVID-19 pandemic and its psychological and health-related consequences. These strategies should consider specific risk factors and follow general public health policies and strategies. Because the association between subjectively assessed low health status and low life satisfaction seems to be universal, public health policy should primarily focus on supporting people with the worst levels of perceived physical health. Further cross-national research should be performed to examine whether the strong association between physical health and life satisfaction is related to the university student population, either in early or throughout adulthood, or the coronavirus pandemic.

This study has several practical implications. To prevent a decrease in life satisfaction during the COVID-19 pandemic, universities should implement various methods to improve technical skills to cope with new technologies, and psychological skills should be taught to allow students to cope with stress effectively. Gori et al. [90] recently showed that a coping with stress, positive attitude, and mature defenses mediate the relationship between perceived stress and life satisfaction. Recent findings from a study in Turkey indicate that life satisfaction could significantly predict resilience [91]. Tomaszek et al. [63] suggested that post-coronavirus therapy should be focused on fundamental existential questions and the meaning of life, since the severity of traumatic sensations may affect the relationship between life satisfaction and post-traumatic growth. An internet-delivered physical activity intervention could be implemented, as shown by an example from Slovenia [92, 93], together with psychological counseling for those university students who suffer from anxiety and depression [94, 95]. Lesser and Nienhuis [49] postulated that health promotion should be targeted at physically inactive people to improve their well-being. University students should be encouraged to exercise at home with limited space to maintain a generally healthy lifestyle during periods of prolonged quarantine [38, 51]. The present study suggests that PA should be recommended, particularly among university students and particularly among those from Turkey, Russia, the Czech Republic, Poland, and Israel, as it is a relatively inexpensive means of increasing the ability of body's immune system to fight the coronavirus.

## Supplementary Information

**Additional file 1.**

**Additional file 2.**

**Additional file 3.**

## Data Availability

This study is a part of an international research project: Well-being of undergraduates during the COVID-19 pandemic: International study [69], registered at the Center for Open Science (OSF) 10.17605/OSF.IO/Q5F4E. The datasets used and/or analysed during the current study are available from the corresponding author on reasonable request.

## List of abbreviations

SARS-Cov-2: severe acute respiratory syndrome-coronavirus-2
COVID-19: coronavirus disease
PA: physical activity
IRB: *Institutional Review Board*
APA: American Psychological Association
EUR: Euro
OSF: Center for Open Science
SWLS: Satisfaction with Life Scale
PNIC: perceived negative impact of coronavirus on students' well-being
GSRH: Generalized Self-Reported Health
SF-12V: 12-Item Short Form *Health* Survey
CI: confidence interval
ANOVA: analysis of variance
HSD: Tukey's honest significant difference
OR: odds ratio
AOR: adjusted odds ratio
