# Supplementary material for: Satisfaction with life among university students from nine countries: Cross-national study during the first wave of COVID-19 pandemic
Source: BMC Public Health. 2021 Dec 11;21:2262. doi: 10.1186/s12889-021-12288-1 (PMC8665700; doi:10.1186/s12889-021-12288-1)
Supplement: Supplementary file 1 — Additional file 1. [file 12889_2021_12288_MOESM1_ESM.pdf]

МІНІСТЕРСТВО ОСВІТИ І НАУКИ УКРАЇНИ
Львівський державний університет фізичної культури імені Івана Боберського

вул. Костюшка, 11, м. Львів, 79007, тел: (032)255-32-01, факс: (032)255-32-08
E-mail: info@ldufk.edu.ua Код ЄДРПОУ 34606048

_____________ № __________ На № ___________________

The study regarding analysis wellbeing during the COVID-19 pandemic is held after consideration by the Bioethics Committee of Lviv State University of Physical Culture and the adoption of a positive decision (protocol number 6 (2020-16-12).

According to the purpose of research, a survey at universities during the academic year 2020/2021 was planned. Written informed consent for participating is obtained from every person and is kept by Prof. Iu. Pavlova. Individuals that are willing to take part in survey fill out questionnaire that is completely anonymous. All procedures performed in studies involving human participants were following the ethical standards of the research committee and with the 1964 Helsinki declaration and its later amendments, and relevant institutional and national research committee ethical standards.

Head of the Bioethics Committee Dr. Y. Boretsky

Vice-rector in Scientific Affairs and External Relations Prof. A. Vovkanych

МІНІСТЕРСТВО ОСВІТИ І НАУКИ УКРАЇНИ * ЛЬВІВСЬКИЙ ДЕРЖАВНИЙ УНІВЕРСИТЕТ ФІЗИЧНОЇ КУЛЬТУРИ * ІМЕНІ ІВАНА БОБЕРСЬКОГО № 34606048
