# Supplementary material for: Satisfaction with life among university students from nine countries: Cross-national study during the first wave of COVID-19 pandemic
Source: BMC Public Health. 2021 Dec 11;21:2262. doi: 10.1186/s12889-021-12288-1 (PMC8665700; doi:10.1186/s12889-021-12288-1)
Supplement: Supplementary file 3 — Additional file 3. [file 12889_2021_12288_MOESM3_ESM.docx]

**אישור ועדת האתיקה במחקר המסלולית לביצוע המחקר**

לכבוד

ד"ר ג'וי בנטוב (חוקרת ראשית)

לאחר עיון במסמכים ובחומרים שהוגשו לוועדה, שוכנענו שהמחקר שבו מעורבים בני אדם, אשר פרטיו מופיעים להלן, אינו ניסוי רפואי, ושהוא עומד בתנאים המפורטים בנוהל אישור מחקר שבו מעורבים בני אדם (ושאינו ניסוי רפואי) וכי ניתן לאשרו לביצוע במסגרת המוצעת.

.

1. מספר הפנייה: 0128-2020
2. שם החוקרים הראשיים: ג'וי בנטוב
3. שם המחקר: רווחה נפשית בקרב סטודנטים בצל הקורונה
4. תוקף האישור : אין הגבלה
5. תנאים והגבלות: אין הגבלה.

כל שינוי, תוספת או סטייה מתוכנית המחקר, טעון אישור בכתב של הוועדה.

על החוקר הראשי במחקר לדווח לוועדה על הפסקת המחקר.

חודשיים בטרם חלוף התקופה המאושרת לביצוע המחקר חובה על החוקר הראשי להעביר לוועדה דוח התקדמות על מהלך המחקר. הוועדה תודיע על החלטתה לגבי המשך המחקר ובהתאם יונפק אישור חדש למחקר.

בתום המחקר יגיש החוקר הראשי לוועדה דוח מסכם על מהלך המחקר ותוצאותיו.

האישור ניתן לחוקר הראשי ואינו ניתן להעברה לאחר.

שם יו"ר הוועדה: ד"ר נילי קרקו-אייל

חתימת יו"ר הוועדה:
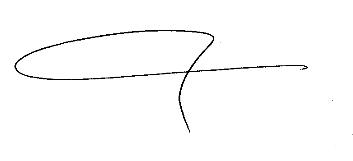


31.5.20
